# Gut Microbiome and Metabolome Variations in Self-Identified Muscle Builders Who Report Using Protein Supplements

**DOI:** 10.3390/nu14030533

**Published:** 2022-01-26

**Authors:** Lauri O. Byerley, Karyn M. Gallivan, Courtney J. Christopher, Christopher M. Taylor, Meng Luo, Scot E. Dowd, Gregory M. Davis, Hector F. Castro, Shawn R. Campagna, Kristin S. Ondrak

**Affiliations:** 1 Sports and Health Sciences, School of Health Sciences, American Public University System, Charles Town, WV 25414, USA; kgallivan@apus.edu (K.M.G.); kristin.ondrak@mycampus.apus.edu (K.S.O.); 2 Department of Physiology, Louisiana State University Health Sciences Center, New Orleans, LA 70112, USA; 3 Department of Chemistry, University of Tennessee at Knoxville, Knoxville, TN 37996, USA; cleathe3@vols.utk.edu (C.J.C.); hcastrog@utk.edu (H.F.C.); campagna@utk.edu (S.R.C.); 4 Department of Microbiology, Immunology, and Parasitology, Louisiana State University Health Sciences Center, New Orleans, LA 70112, USA; ctay15@lsuhsc.edu (C.M.T.); mluo2@lsuhsc.edu (M.L.); 5 Molecular Research LP, 503 Clovis Rd, Shallowater, TX 79363, USA; sdowd@mrdnalab.com; 6 Kinesiology and Health Studies, Southeastern Louisiana University, Hammond, LA 70401, USA; gregory.davis-3@selu.edu; 7 Biological and Small Molecule Mass Spectrometry Core, University of Tennessee at Knoxville, Knoxville, TN 37996, USA

**Keywords:** gut microbiota, gut microbiome, resistance training, strength training, dietary protein, gut metabolome, nitrogen metabolism

## Abstract

Muscle builders frequently consume protein supplements, but little is known about their effect on the gut microbiota. This study compared the gut microbiome and metabolome of self-identified muscle builders who did or did not report consuming a protein supplement. Twenty-two participants (14 males and 8 females) consumed a protein supplement (PS), and seventeen participants (12 males and 5 females) did not (No PS). Participants provided a fecal sample and completed a 24-h food recall (ASA24). The PS group consumed significantly more protein (118 ± 12 g No PS vs. 169 ± 18 g PS, *p =* 0.02). Fecal metabolome and microbiome were analyzed by using untargeted metabolomics and 16S rRNA gene sequencing, respectively. Metabolomic analysis identified distinct metabolic profiles driven by allantoin (VIP score = 2.85, PS 2.3-fold higher), a catabolic product of uric acid. High-protein diets contain large quantities of purines, which gut microbes degrade to uric acid and then allantoin. The bacteria order Lactobacillales was higher in the PS group (22.6 ± 49 No PS vs. 136.5 ± 38.1, PS (*p =* 0.007)), and this bacteria family facilitates purine absorption and uric acid decomposition. Bacterial genes associated with nucleotide metabolism pathways (*p <* 0.001) were more highly expressed in the No PS group. Both fecal metagenomic and metabolomic analyses revealed that the PS group’s higher protein intake impacted nitrogen metabolism, specifically altering nucleotide degradation.

## 1. Introduction

Individuals striving to build muscle often combine resistance training with increased protein intake. A recent review article reported that 40–100% of athletes used some type of supplement, including protein, intending to improve athletic performance [1]. Most of the protein-supplement products on the market are whey-, casein-, or soy-based, and research has shown that whey, casein, and/or soy protein promote/s similar amounts of protein synthesis and strength gains [2].

In the last few decades, the importance of gut microbiota in promoting health has been recognized. On average, the human gastrointestinal tract harbors from 200 to more than 1000 species of bacteria [3,4]. The largest concentration of microbes, including bacteria, archaea, fungus, and viruses, is found in the colon. Food has the biggest impact on shaping the gut microbiome, although many other daily activities, such as exercise and sleep, can also affect the relative abundance of microbes. A shift in the relative abundance of even the microbes that sparsely populate the gut can be beneficial or harmful to one’s health.

Recent studies support the idea that acute and chronic exercise alters the gut microbial environment, enhancing the growth of beneficial microbes. However, most of the research has focused on aerobic exercise. For example, Estaki et al. [5] showed that cardiorespiratory fitness was positively associated with microbial diversity; in other words, the more fit an individual, the more diverse their gut microbiota. Moreover, the increased diversity favored an increased relative abundance of microbial species that produce butyrate. Clarke et al. found greater diversity within the Firmicutes phylum in elite rugby players, and many species in this phylum produce butyrate [6]. These two studies demonstrate that aerobic exercise is associated with increased gut microbial diversity, and increased microbial diversity is associated with improved health [6].

Only a few studies have examined the effect of resistance exercise on the gut microbiome. Bycura et al. [7] investigated the impact of an eight-week intervention of cardiorespiratory or resistance exercise on the gut microbiome. They found few gut microbiota changes before and after training in either group. Participants in the resistance-training group with the biggest microbiome changes did not necessarily have the largest performance gains [7]. Unfortunately, diet and supplement use were not reported, so their influence on exercise gains or microbiota diversity cannot be ascertained. In another study, Jang et al. [8] compared sedentary men, bodybuilders, and distance runners. They found that each athlete type had a specific diet pattern; for example, bodybuilders consumed a high-protein, low-carbohydrate diet, while distance runners consumed a high carbohydrate diet. Alpha and beta microbiome diversity did not differ by athlete type, but there were differences in gut microbiota taxa at the genus and species level.

In addition to the effect of exercise on the gut microbiome, exercise also increases mitochondrial biogenesis in different tissues, including muscle [9]. Recently, it was demonstrated that traditional high-load resistance training could stimulate muscle mitochondrial biogenesis and mitochondrial respiratory function [10]. The gut microbiota has also been shown to regulate critical transcriptional co-activators, transcription factors, and enzymes involved in mitochondrial biogenesis, such as PGC-1alpha, SIRT1, and APMK genes [11]. It appears that this is mediated through signaling from the gut microbiota to mitochondria. Several studies suggest that the metabolites produced by commensal gut microbiota, such as short-chain fatty acids, might play a role [11]. Butyrate is one of the short-chain fatty acids produced. Interestingly, when mice were given a dietary supplement containing butyrate, it led to enhanced mitochondrial biogenesis [12].

Studies investigating the effect of probiotic ingestion on gut health and exercise performance offer further support that the gut may play a role in muscle building. For example, Chen et al. [13] supplemented mice with *Lactobacillus plantarum* TWK10 and found that six weeks of supplementation increased muscle mass and muscle-mass grip strength. This particular bacterium is a lactic acid producer and is commonly found in many fermented foods and anaerobic plant matter [14].

Finally, a meta-analysis of protein supplementation and resistance training showed that dietary protein supplementation was significantly associated with increased strength [15]. Lending further support to this relationship, Cronin et al. [16] showed participants who consumed whey protein for 8 weeks, combined with resistance training, experienced a marked alteration in the diversity of their gut virome. Interestingly, participants who underwent 8 weeks of combined aerobic and resistance training but were otherwise sedentary only modestly changed the composition and activity of the gut microbiome.

Therefore, this exploratory cross-sectional study sought to identify the bacteria and metabolites present in the fecal matter of self-identified muscle builders who did or did not report taking a protein supplement (PS vs. No PS, respectively). We hypothesized that our group reporting protein-supplementation usage would have a different gut microbiota and metabolic profile than those who reported no protein supplement usage.

## 2. Materials and Methods

This exploratory cross-sectional study aimed to identify the bacteria present in the fecal matter of self-identified muscle builders using resistance exercise who reported using or not using a protein supplement. Participants were recruited from Sports and Health Science majors at the online university, American Public University System (APUS). This study was approved by the Institutional Review Board at APUS (protocol # 2018-097) and the Louisiana State University Health Sciences Center (LSUHSC) (protocol # 10217).

### 2.1. Experimental Approach

**Figure 1:**
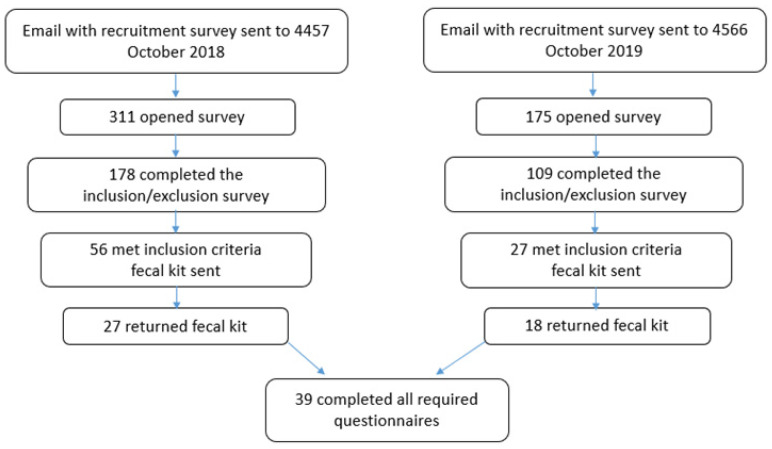
Flowchart showing participant recruitment.

A schematic for recruiting participants is shown in Figure 1. Participants were excluded if they met at least one of the following criteria: (1) took an antibiotic in the last three months, (2) consumed an anti-diarrhea medicine in the last week, (3) took a laxative in the last week, (4) consumed prebiotics in the last week, (5) consumed probiotics in the last week, (6) were diagnosed with cancer, (7) have Crohn’s disease, (8) were taking prescription drugs other than oral contraceptives, (9) were cutting for an upcoming competition or tournament, (10) were under 25 years of age, or (11) lived outside the contiguous United States. We asked potential participants to share their maximum squat, bench press, or deadlift and then calculated a weight-lifted-to-body-mass ratio. Those who could not lift at least half of their body weight were excluded. Each participant was asked if they used protein supplements so they could be assigned to one of two groups: protein supplement (PS) and no protein supplement (No PS). The participants that met the criteria were sent an email welcoming them to the study and requested verification that they were willing to participate. If their response was positive, they were sent a fecal collection kit. After collecting their fecal sample, the participant completed a supplement questionnaire; a workout questionnaire; an online version of the International Physical Activity Questions (IPAQ); and a 24-h food recall, using the self-administered 24-h dietary assessment tool (ASA24). Each participant completed the study at his or her physical location anywhere in the contiguous United States. Participants were recruited in two cohorts one year apart at the same time of year (October/November/December). For successful participation in the study, the participant had to (1) complete a signed written informed consent, (2) complete a signed HIPAA consent, (3) acknowledge that they were training to build muscle, (4) complete the required questionnaires, and (5) return the fecal sample. Thirty-nine participants provided a fecal sample and completed all the required forms.

### 2.2. Sample Collection

A fecal sample was collected by using an at-home, self-collection kit (The Biocollective, Denver, CO, USA) sent to participants via Fed Ex overnight, along with a freezer brick, Styrofoam shipping container, and cardboard box to fit over the Styrofoam shipping container. The kit contained a specially designed biocollector that was placed over the toilet for fecal collection. Once the fecal sample (without urine) was collected, the biocollector was inserted into a special zip-locking barrier bag that was inserted into a zip-locked specimen bag. Then the specimen bag was placed in a Styrofoam shipping container, and a frozen freezer brick was placed on top. The Styrofoam container was placed into the cardboard box, sealed, and shipped overnight. To prevent receiving samples on a Saturday or Sunday, the participant was instructed to ship the sample on the same day of collection and collect it on Monday–Thursday, no holidays. If the sample was not returned within 24 h of collection, the sample was discarded, and the participant was excluded from the study. Upon receipt in the lab at LSUHSC, the sample was mixed in the sample bag in a biosafety level-2 hood and small portions aliquoted into sterile microcentrifuge tubes designed for cold storage. The tubes were placed in liquid nitrogen for quick freezing and stored in a −80 °C freezer. At the same time, a sample of approximately 250 mg was processed for DNA isolation, using the QIAamp PowerFecal DNA Kit (Qiagen, Germantown, MD, USA), which included bead-beating. The isolated DNA was stored at −80 °C until microbial community analysis.

### 2.3. Dietary Assessment

Participants were asked to recall the foods they ate 24 h prior to their fecal sample collection. They used the freely available, automated, web-based, self-administered 24-h dietary assessment (ASA24) (https://epi.grants.cancer.gov/asa24/, accessed on 6 June 2021) [17], which is available on the web and mobile devices and is funded and maintained by the National Cancer Institute. Each participant was sent a unique user ID and password. Although the ASA24 program collects information on supplement use, we also asked participants to provide information about their supplements on a separate form. The 2010–2015 Healthy Eating Index (HEI) was calculated from the food group data provided by ASA24, using the SAS program code available for free, online, from the National Cancer Institute (https://epi.grants.cancer.gov/hei/calculating-hei-scores.html, https://epi.grants.cancer.gov/asa24/, accessed on 6 June 2021).

### 2.4. Physical Activity Assessment

Physical activity was assessed by using the International Physical Activity Questionnaire (IPAQ) long-form converted to an online format. Moreover, each participant was asked to provide their muscle-building workout routine on a separate form, since the IPAQ does not ask specifically about resistance training. In this form, participants reported the exercise name, workouts per week, sets per workout, reps per set, weight lifted, and 1-RM. Volume loads (weight lifted in kg x reps x sets x workouts per week) for upper-body or lower-body exercises were calculated similarly to the methods of Haff [18].

### 2.5. Microbial Community Analysis

A Thermo Scientific™ NanoDrop™ spectrophotometer (ThermoFisher Scientific, Waltham, MA, USA) was used to determine the quantity and purity of isolated fecal DNA. Two amplification steps, using the AccuPrime Taq high fidelity DNA polymerase system (Invitrogen, Carlsbad, CA, USA), were performed to prepare a sequencing library. Moreover, a negative control with the control from DNA extraction and a positive control, using the Microbial Mock Community HM-276D (BEI Resources, Manassas, VA, USA), were included in the amplicon library preparation. Genomic DNA and gene-specific primers with Illumina adaptors were used to amplify the 16S ribosomal DNA hypervariable region V4: forward 5′TCGTCGGCAGCGTCAGATGTGTATAAGAGACAG GTGCCAGCMGCCGCGGTAA3′; reverse 5′ GTCTCGTGGGCTCGGAGATGTGTATAAGAGACAG GGACTACHVGGGTWTCTAAT 3′. AMPure XP beads were used to purify the indexed amplicon libraries and quantified by using Quant-I PCR products. The purified amplicon DNA was amplified by using primers with different molecular barcodes:

forward 5’ AATGATACGGCGACCACCGAGATCTACAC [i5] TCGTCGGCAGCGTC 3′; reverse 5’ CAAGCAGAAGACGGCATACGAGAT [i7] GTCTCGTGGGCTCGG 3′.

The indexed amplicon libraries were purified by using AMPure XP beads, quantified by using Quant-iT PicoGreen (Invitrogen), and then normalized and pooled. The KAPA Library Quantification Kit (Kapa Biosystems, Cape Town, South Africa) was used to quantify the pooled library, which was diluted and denatured according to Illumina’s sequencing library preparation guidelines. As an internal control and to increase 16S rRNA amplicon library diversity, 10% PhiX was added to the sequencing library. The paired-end sequencing was performed on an Illumina MiSeq (Illumina, San Diego, CA, USA) using a 2 × 250 bp V2 sequencing kit.

QIIME2 (Quantitative Insights Into Microbial Ecology) with the DADA2 plugin [19] was used to process raw fastq files. Forward and reverse reads were truncated to a uniform length of 240 bp, and 20 bp were trimmed off the front of each read to remove the primer. DADA2 identified amplicon sequence variants (ASVs) were merged, and any that ranged outside of the expected 250–255 bp amplicon length were discarded. Any ASVs that appeared in only one sample were removed by using contingency-based filtering, and chimeric ASVs were removed by using the consensus method. ASVs were aligned by using mafft and fasttree [20], and a phylogenetic tree for diversity analysis was built. Greengenes v13.8 was used for taxonomic classification [21]. After primary data analysis, the remaining reads were analyzed by using QIIME2 [3].

The QIIME analysis included 39 samples, with read counts ranging from 6729 to 99,983, with an average read count per sample of 44,812. Alpha rarefaction was performed at a level of 6729 reads to include all samples.

### 2.6. Prediction of Metabolic Profile

The 16S sequencing data were used to identify potential microbial functions. The raw data were formatted and imported into QIIME2. Closed-reference clustering against the Greengenes 13_5 97% OTUs reference database was used to develop a de-replicated feature table and representative sequences. The closed-reference OTU table was used as input into the PICRUSt [22] pipeline, and the resulting PICRUSt metagenome data were further analyzed by using STAMP (Statistical Analysis of Metagenomic Profiles) [23]. Pathways were labeled at Level 2, since several pathways were not classified at Level 1, which causes an error in STAMP. From this, KEGG (Kyoto Encyclopedia of Genes and Genomes) pathways were compared between protein-supplemented (PS) and non-protein-supplemented (No PS) participants.

### 2.7. Metabolomics

Samples were processed at the Biological and Small Molecule Mass Spectrometry Core (BSMMSC) at the University of Tennessee, Knoxville, TN (RRID: SCR_021368). The fecal sample from each participant was divided into aliquots (roughly 50–100 mg) and extracted in biological triplicate. Polar metabolites were extracted from fecal samples using an acidic acetonitrile extraction procedure, using 40:40:20 HPLC grade ACN/MeOH/H_2_O with 0.1 M formic acid. [24] Global metabolic profiling, using ultra high-performance liquid chromatography-high-resolution mass spectrometry (UHPLC-HRMS), was used to analyze the fecal microbiome. A 25-min reverse-phase chromatographic separation was performed by using a Synergi 2.6 µm Hydro RP column (100 mm × 2.1 mm, 100 Å; Phenomenex, Torrance, CA, USA) and an UltiMate 3000 pump (Dionex). After chromatic separation, analytes were ionized by using negative-mode electrospray ionization. A Thermo Scientific Exactive Plus Orbitrap (San Jose, CA, USA) was utilized for mass analysis, operating in full-scan mode [25,26]. A package from ProteoWizard, msConvert, was used to convert raw spectral files to mzML format [27]. Metabolomics analysis and visualization engine (MAVEN) was used to identify metabolites based on exact mass (±5 ppm) and retention time, using an in-house library [28,29]. A total of 170 metabolites were identified from the untargeted metabolomics analysis.

### 2.8. Statistical Analysis

A power calculation determined that 24 participants were needed per group (PS and No PS). Thirty-nine participants completed the study prior to the beginning of the COVID-19 pandemic. We decided to stop participant recruitment at the start of the pandemic since its duration was unknown, and we did not want to risk exposure to the virus from fecal samples.

Data were expressed as mean ± SEM (standard error of the mean), and the tables presented the actual *p*-value. A value of *p <* 0.05 was considered statistically significant and was determined a priori. SPSS (IBM Corp. Released 2020. IBM SPSS Statistics for Windows, Version 27.0. IBM Corp: Armonk, NY, USA) was used for statistical comparisons. Differences in alpha and beta diversity were determined by using QIIME 1.9.0, and figures were drawn by using GraphPad Prism 9 (GraphPad Software, San Diego, CA, USA). Significant differences in bacterial species between the No PS and PS groups were determined by using Mann–Whitney U. Benjamini–Hochberg was applied to decrease the false discovery rate. Only *p*-values < 0.01 and significant following the FDR correction are reported for the bacterial species identified as significantly different.

STAMP was used to determine statistical differences in functional pathways between the groups and to generate post hoc (Tukey–Kramer) plots for each KEGG pathway that was significantly different between No PS, and PS. Bonferroni was used to correct for multiple analyses.

Metabolite peak intensities were normalized by the weight of each fecal sample aliquot. MetaboAnalyst 5.0 was used to generate partial-least-squares discriminant analysis (PLS-DA) plots, variable importance in projection (VIP) scores, and pathway analysis using the normalized metabolomics data. In MetaboAnalyst, the normalized data were filtered by interquartile range, log-transformed, and Pareto scaled [30]. Heatmaps were generated by using R (version 1.0.153), which displayed log_2_ fold changes and *p*-values calculated by a Student’s *t*-test.

## 3. Results

Participant characteristics are shown in Table 1. There were no significant differences in age, weight, height, or BMI between the PS and No PS groups. Seventy-one percent of the No PS group was male, while 64% of the PS group was male.

The participants self-identified as muscle builders. As part of the selection criteria, each potential participant provided the maximum weight he or she could lift for the squat, bench press, or deadlift. On average, both groups could lift 1.9 ± 0.2 lift weight (lbs)/body weight (lbs); (range of 1.0 to 3.6 lbs/lbs). Seventeen participants reported how long they had been lifting, which averaged 11 ± 2 yrs. The data collected from the training worksheet confirmed that our participants participated in activities designed to maintain or increase their current muscle mass specifically. Moreover, this information was compiled and reported as upper and lower body resistance exercise volume (Table 1).

The participant’s physical activity was assessed by using the IPAQ. There was no significant difference in the amount of time the participants spent sitting daily, although the PS group tended to sit less (No PS: 324 ± 250; PS: 267 ± 124 min/day). As for physical activity, total walking (No PS: 1893 ± 432; PS: 2318 ± 544 METS-min/week) and moderate activity (No PS: 2613 ± 645; PS: 3432 ± 733 METS-min/week) did not differ significantly between the two groups. Both groups’ total physical activity level was consistent with a high level of physical activity (vigorous activity > 1500 MET minutes per week). Still, total physical activity was significantly higher for the PS group, because they spent significantly more time doing vigorous activity.

Twenty-four participants (65%) reported using a supplement (not protein), and 41 different supplements were reported. The most commonly used supplement was creatine (13.8%), and the supplements that four or more participants used were AminoX branch chain amino acids by BSN (7.5%), a multivitamin pill (6.3%), beta-alanine (5%), fish oil (5%), and glutamine (5%).

Dietary intake is reported in Table 2. There were no significant group differences in energy intake, but the macronutrient distribution of the calories from protein, and fat was significantly different. The No PS group consumed significantly more fat than the PS group (Table 1). Because the percent of calories derived from carbohydrates was similar between the groups (Table 1), the difference was made up with protein. The PS group derived more calories from protein (28 ± 2%), while the No PS derived 20 ± 2% of their calories from protein (*p <* 0.0027). This marked difference in protein intake reflected a difference of more than 0.5 g/kg of protein intake for the PS group. This protein was primarily derived from food sources, as only 4 of the 22 participants consumed a protein supplement (25.3 ± 3.8 g, 13% of their total protein intake) on the day of the recall. Most of the difference in protein intake between the PS and No PS was derived from beans and peas. The PS group was asked to report the protein supplement they typically consumed. Twenty-one different supplements were reported, and 17 (81%) contained whey protein.

Despite a similar carbohydrate intake, the PS group consumed about 1.5 times more fiber than the No PS group. Total sugar consumption was not significantly different between the groups, but the No PS group tended to consume more sugar. Finally, alcohol and caffeine intake tended to be higher in the No PS group, although not significant. Thirty-five percent of the No PS group consumed alcohol, while 14% of the PS group did. As for caffeine, 70% of the No PS and 77% of the PS group consumed a beverage that contained caffeine.

The 2010–2015 HEI index, a value that determines how closely one’s diet adheres to the US Dietary Guidelines, is 59 for the American diet (https://www.fns.usda.gov/healthy-eating-index-hei, 16 November 2021). A score of 100 indicates that one is adhering to the guidelines. The average score for both the No PS and PS groups was close to 59 (Table 2); the No PS group had a lower HEI score than the PS group, although not significant.

**Figure 2:**
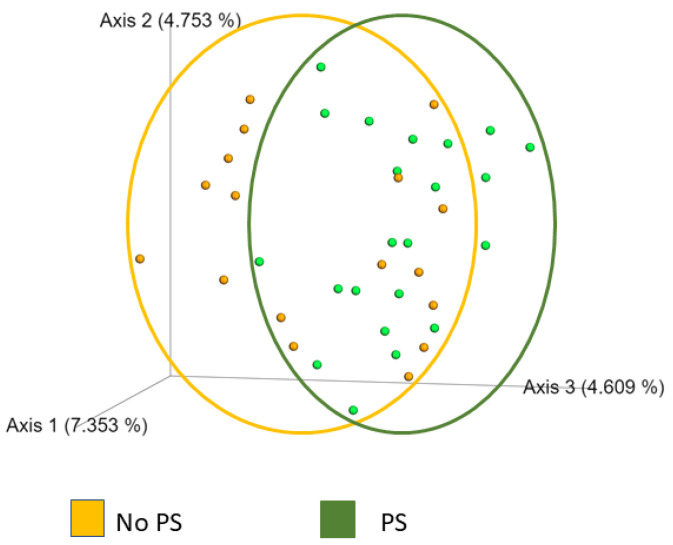
Jaccard plot of beta diversity. Each orange dot corresponds to a participant (No PS) who reported not using protein supplements. The orange circle highlights the range of values. Each green dot represents one participant who reported using protein supplements (PS). The green circle shows the variation. Although the groups overlap, the differences were significant (*p =* 0.035), demonstrating community separation by this method.

Bacterial diversity was measured within a sample (alpha) and between samples (beta). We used Chao, Shannon, Faith PD, and Simpson to measure alpha diversity. These indexes consider the number of unique operational taxonomic units (OTUs), richness, the relative abundance of OTUs, and evenness. All four measures found that the No PS and PS groups had similar alpha diversity. Bray–Curtis, Jaccard, and UniFrac were used to measure beta diversity. Only Jaccard similarity (ANOSIM) was significantly different (*p =* 0.035). As shown in Figure 2, the two groups overlapped with a modest difference in community separation between the two groups.

Table 3 shows the bacterial species that were significantly different between the No PS and PS groups. In all cases, a greater relative abundance of the taxa was observed in the PS group. As expected, the changes occurred in the more predominant bacteria phyla, Bacteroidetes, Firmicutes, and Actinobacteria, which made up 46%, 43%, and 2.5% of the bacteria present in the gut, respectively. The most changes in taxa were observed in the Firmicutes phylum (74%). The significantly different changes observed in Actinobacteria derived from the Coriobacterilia class with *Adlercreutzia* at the genus level. For the Bacteroidetes phyla, the changes were seen in the Baceroidales order at the family or genus level. Five different families within the Firmicutes were affected. The most striking difference was with the bacteria *Ruminococcaceae* family, 34 times greater in the PS group than the No PS group.

The bacteria identified as significantly different were correlated with dietary protein, fiber, and fat. These associations are shown in Table 3, and the bacteria species belonged to the Actinobacteria, Bacteroidetes, and Firmicutes phyla. All correlations were positive, with more bacteria being associated with dietary fat than protein or fiber. Although dietary fat was not significantly different between the groups, 41% of the No PS dietary calorie intake came from fat, while 34% of the PS dietary calories were derived from fat. Only two bacterial species were correlated with dietary fiber (not shown in table): p__Firmicutes;c__Clostridia;o__Clostridiales;f__Lachnospiraceae;g__Coprococcus (*p <* 0.032) and p__Firmicutes;c__Clostridia;o__Clostridiales;f__Ruminococcaceae;g__Oscillospira (*p <* 0.032).

**Figure 3:**
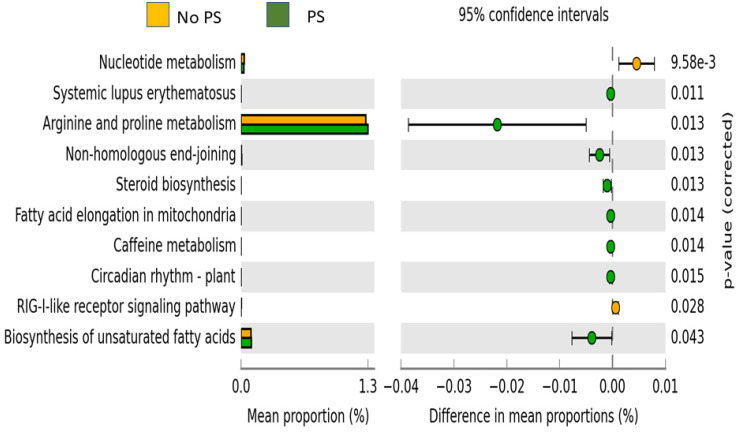
Predicted functional pathways. Pathways that were significantly different between the two groups are shown along with the Bonferroni-corrected *p*-value. Orange bars are the No PS group, while the green bars are the PS group.

The predicted functional pathways that were significantly different are shown in Figure 3. Ten pathways emerged as significantly different between the No PS and PS groups. Eight of the ten pathways (arginine and proline metabolism, biosynthesis of unsaturated fatty acids, caffeine metabolism, circadian rhythm-plant, fatty acid elongation in mitochondria, non-homologous end-joining, steroid biosynthesis, and systemic lupus erythematosus) were more highly expressed in the PS group. The nucleotide metabolism and RIG-1-like receptor signaling pathway were more highly expressed in the No PS samples.

**Figure 4:**
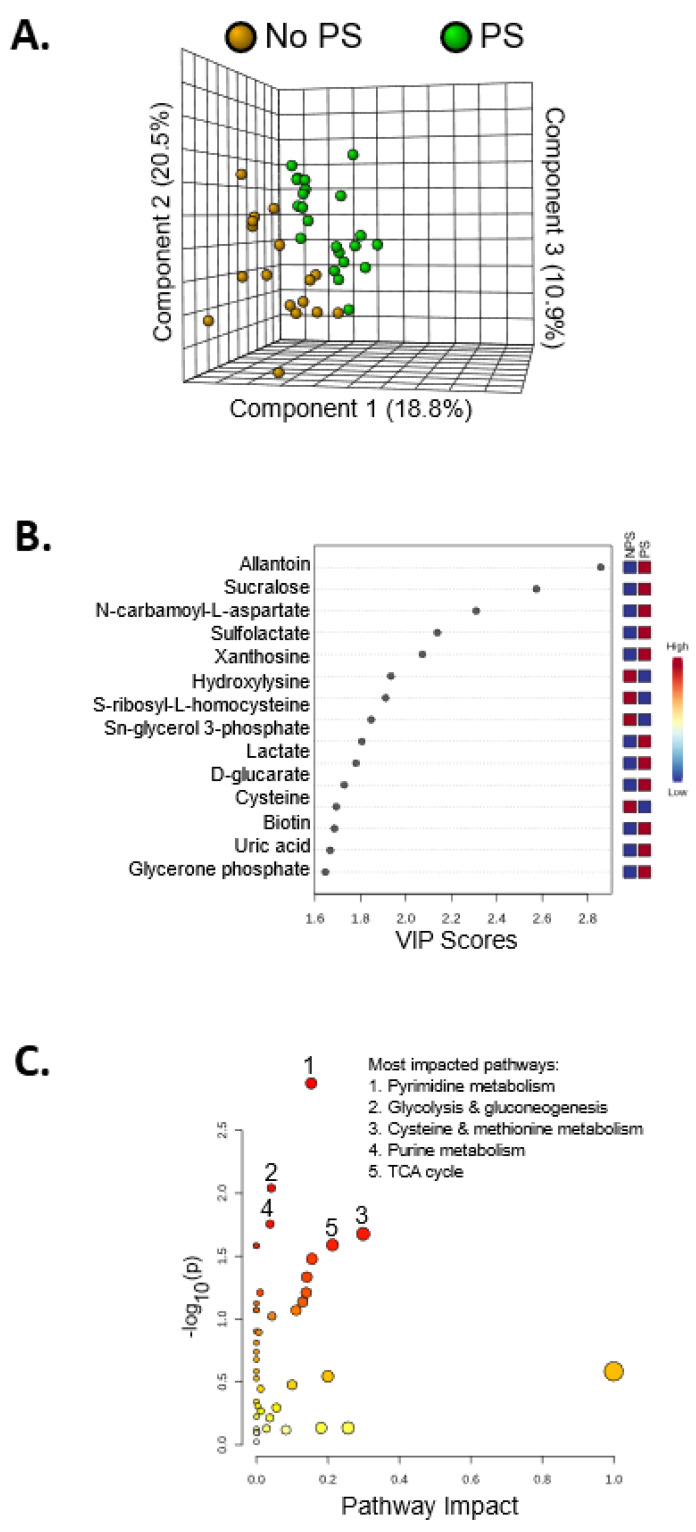
Metabolomics analysis showing PS induces metabolic alterations. (**A**) 3D PLS-DA plot showing separation of the PS and NPS groups, indicating unique metabolic profiles. (**B**) This plot shows the top 15 metabolites with the highest VIP scores contributing most to the observed separation of groups. Allantoin contributes most to the separation of the PS and NPS groups in the PLS-DA plot. (**C**) Metabolites with a VIP score > 1 were used for pathway analysis. The pathways impacted most by PS include pyrimidine metabolism, glycolysis and gluconeogenesis, cysteine and methionine metabolism, purine metabolism, and the TCA cycle.

**Figure 5:**
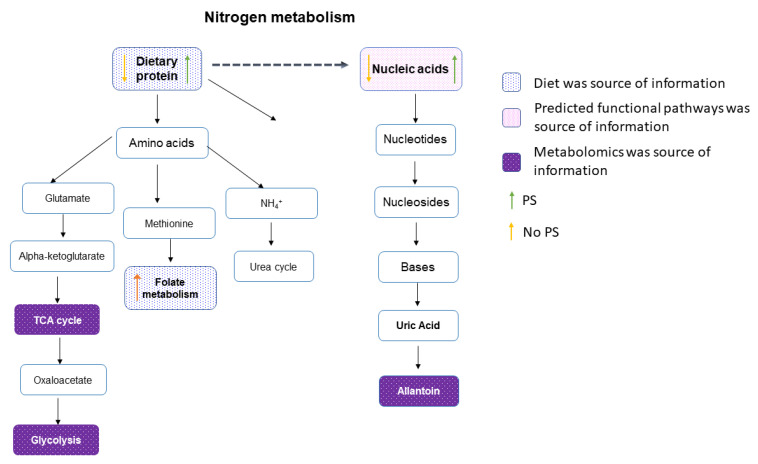
Nitrogen metabolism pathways. Assembling the metabolome and microbiome data suggested nitrogen metabolism was altered in the gut environment of participants who reported consuming a protein supplement compared to those who did not. Blue boxes represent information derived from the participant’s dietary recall. The orange and green arrows in each box show which group had higher or lower intake. Evidence derived from predicted functional pathways is highlighted in pink, while the purple boxes are metabolites or pathways identified by metabolomics.

**Figure A1:**
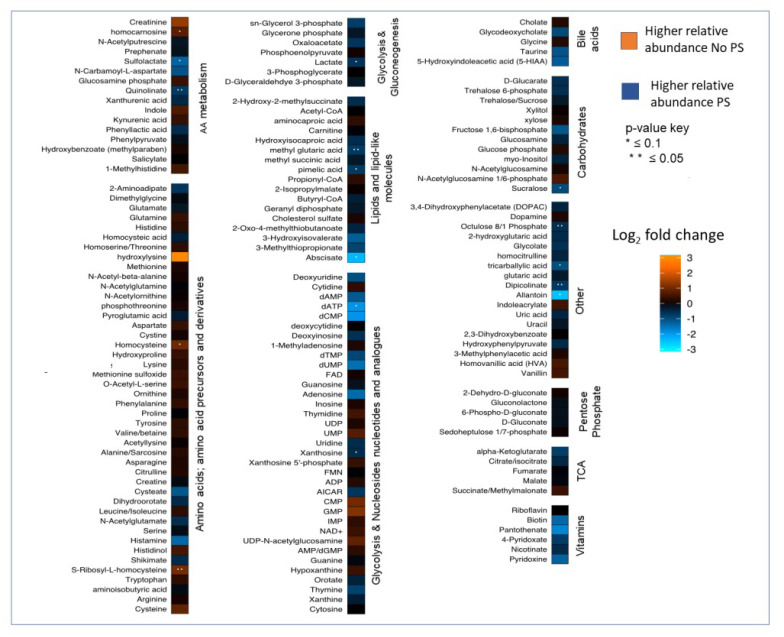
Heatmap showing the log_2_ fold change and *p*-values for all identified metabolites in fecal samples. Metabolite relative abundances were altered by PS.

Alterations in the fecal metabolome are shown in Figure 4. Heatmaps serve as a visualization tool for changes in the relative abundance of metabolites (Figure A1). A few metabolites were significantly different in relative abundance between the two groups (Figure 4A): allantoin (*p <* 0.01), dipicolinate (*p <* 0.05), tricarballylic acid (*p <* 0.01), octulose 8/1 phosphate (*p <* 0.05), xanthosine (*p <* 0.01), dATP (*p <* 0.01), abscisate (*p <* 0.01), pimelic acid (*p <* 0.01), methyl glutaric acid (*p <* 0.05), lactate (*p <* 0.01), sucralose (*p <* 0.01), S-ribosyl-L-homocysteine (*p <* 0.05), homocysteine (*p <* 0.01), quinolinate (*p <* 0.05), sulfolactate (*p <* 0.01), and homocarnosine (*p <* 0.01). Despite few significant changes, the 3D PLS-DA showed separation of the PS group and the No PS group, indicating different metabolic profiles between the groups (Figure 4A). Metabolites with a VIP score > 1 drove the separation in the PLS-DA plot, contributing most to the differences observed between groups (Figure 4B). Using metabolites with a VIP score > 1 for pathway analysis, it was evident that the purine and pyrimidine metabolism were altered in response to protein supplementation. Additionally, glycolysis, cysteine and methionine metabolism, and the TCA cycle were also perturbed (Figure 4C). All of the altered pathways are interconnected with nitrogen metabolism (Figure 5).

## 4. Discussion

To our knowledge, this is the first study to investigate the gut microbiome of self-identified muscle builders who reported using protein supplementations. Often, a high-protein, low-dietary-fiber diet is associated with bodybuilding. Scientific studies have shown that fiber and protein can influence the abundance of microbial communities present in the gut; however, protein’s effects are less understood than fiber. The recommended fiber intake for men and women under 50 years of age is 38 and 25 g/day, respectively [31]. However, dietary studies have shown that many individuals fall short of these guidelines. For example, in a study comparing the gut microbiota of 15 distance runners and 15 bodybuilders, Jang et al. [8] reported that dietary fiber intake was similar between the two groups: 17 g/day (distance runners) and 19 g/day (bodybuilders). However, the average protein intake was 103 g for the distance runners and 236 g for the bodybuilders. Interestingly, there were differences in the gut microbiome between these groups. At the genus and species levels, *Faecalibacterium*, *Clostridium*, *Haemophilus*, and *Eisenbergiella* were the highest (*p* < 0.05) in bodybuilders, while *Bifidobacterium* and *Parasutterella* were the lowest (*p <* 0.05) in bodybuilders. In our study, fiber intake for our groups was similar to Jang et al.’s [8] study, but, unexpectedly, the PS group consumed 8 gms more fiber than the No PS group. However, neither group met the dietary recommended amount of fiber. Our participants’ average protein intake was less than Jang et al. [8] bodybuilders’ protein intake. *Faecalibacterium* was the only bacteria for which we observed changes that Jang et al. [8] also reported.

The Recommended Dietary Intake for protein is 0.8 g/kg body weight [31] based on net nitrogen balance, translating to a mean dietary need of 63 g/day for our athletes. However, protein is needed to build and repair muscle, so 1.2 to 2.0 g protein/kg body weight/day is recommended for strength-training athletes [32]. Our participants consumed 1.5 to 2.2 g protein/kg body weight, with the No PS group being at the lower end and the PS group at the upper end of the recommendation. On a moderately high protein diet in which protein makes up 15% of total energy intake, 17 g dietary protein/day goes undigested in the small intestine and enters the colon for putrification [33]. In our study, protein intake was 20–27% of calories. Based on Gibson et al.’s [33] findings, we deduct more than 17 g of protein reached the colon for putrification.

A high-protein, low-carbohydrate diet favors a pathogenic pro-inflammatory colonic microbiota. Jang et al. [8] concluded from their study of sedentary individuals, bodybuilders, and distance runners that a high-protein diet might negatively impact gut microdiversity and decrease short-chain fatty acid–producing commensal bacteria. Increased protein fermentation can be attenuated by adding dietary fiber [34]. While our PS group had a higher protein intake, their fiber intake was less than the dietary recommended amount and therefore may not have been sufficient to provide this benefit. We did note an increase in several butyrate-producing species in the gut microbiota of the PS group. We did not measure fecal short-chain fatty acid content to know the amount of butyrate present in the feces.

In addition to protein intake, diet quality affects gut microbiome diversity [35]. HEI is a measure of dietary quality, and both groups’ score demonstrated that they did not meet the US Dietary Guidelines. Thus, only one diversity measure was significantly different between the two groups, so despite the significant difference in protein and fiber intake, these differences were not enough to alter gut microbiota diversity. Other studies have reported no change in gut microbial diversity following resistance exercise [9,36,37].

In healthy individuals, the phyla Firmicutes, Bacteroidetes, Actinobacteria, and Proteobacteria make up 98% of the bacteria present in the human GI tract [36,38]. Our study observed changes in the relative abundance of species within three of these phyla, namely Firmicutes, Bacteroidetes, and Actinobacteria. More changes were seen in the Firmicutes phyla, and many species in this phylum are butyrate-producing [37]. The Lactobacillales abundance was significantly greater in the PS group, and this family of bacteria functions in purine absorption [39] and uric acid decomposition [40].

Host health is affected by the structure and diversity of the gut microbiota. At the genus level, we observed higher levels of Adlercreutzia, Bacteroides, Turicibacter, Anaerostipes, Coprococcus, Faecalibacterium, Oscillospira, Ruminococcus, and Phascolarctobacterium in the protein-supplemented group. Oscillospira is a common gut bacterial genus, and several recent gut microbiota investigations have demonstrated its underlying significance for host health in several chronic diseases, such as obesity [41]. Chen et al. [41] found it inversely correlated with Bristol stool type and that it may play a role in decreased bowel movements and aggravate constipation. Constipation can be a problem with bodybuilders who consume large amounts of protein. However, none of our participants reported constipation, we excluded participants taking laxatives, and the Bristol score was similar between both groups (~3). This score characterizes stool as sausage-like with cracks on the surface. In addition, Oscillospira has also been positively associated with leanness [42]. *Phascolarctobacterium* is a substantial acetate/propionate producer (short-chain fatty acids) and is associated with the metabolic state and positive mood [43]. Jang et al. [8] found Faecalibacterium significantly higher in bodybuilders compared to distance runners and sedentary individuals.

Only a few studies have looked at the effect of protein supplementation, usually whey, on the gut microbiomes of athletes. For example, Moreno-Perez et al. supplemented cross-country runners with 10 g whey isolate and 10 g beef hydrolysate in 200 mL orange drink for 10 weeks. They found an increased abundance of the Bacteriodetes phylum and decreased *Bifidobacterium longum*.

Finally, nitrogen metabolism can be altered by protein consumption. A high-protein diet contains a large quantity of purine, a nucleotide. Uric acid is produced when purines are enzymatically degraded [44]. Uric acid is then further degraded to allantoin, the metabolite with the highest VIP score. The PS group had a significantly higher allantoin relative abundance in their feces compared to the No PS group. Since allantoin and uric acid, along with other metabolites involved in purine metabolism, are driving the separation between the PS and No PS groups, it can be concluded that higher levels of dietary protein favor purine degradation. This evidence is further supported by a greater relative abundance of f. Lactobacillales in the PS group (*p* = 0.007), a bacteria family that functions in purine absorption and uric acid decomposition [45]. Additionally, genes encoding for nucleotide metabolism were significantly more abundant in the No PS group. This observation could be expected, because the No PS group had a lower protein intake, which leads to fewer purines, creating a greater need for genes involved in purine metabolism.

### Limitations

This study is not without its limitations. First, the participants were recruited online, and although we emailed many potential candidates, we demonstrated that participants could be recruited in this manner. Second, once lockdowns from the COVID pandemic started, we could no longer recruit participants, and this reduced our sample size. Third, we had to rely on the participants self-reporting their resistance training, because they were recruited and participated in the study online. Self-reporting has inherent errors, but we used several measures, self-reported workout form and IPAQ, to verify their training claims. These were used to determine if the participants were physically active and had consistent resistance training. Fourth, the two groups were divided based on the participant’s reported protein supplement use. All PS group participants reported using a protein supplement, while the No PS group reported not using a protein supplement to promote muscle gains. We also verified this with the ASA24 24-h recall. The ASA24 reports supplement usage, and only the participants in the protein-supplemented group reported using a protein supplement 24 h before they collected their fecal sample.

## 5. Conclusions

Differences in diet and the gut microbiome and metabolome suggested that protein influenced the gut microbiota. The group reporting protein-supplement usage had more fecal metabolites and bacteria associated with nitrogen breakdown, leading to increased uric acid. In contrast, those participants who did not use a protein supplement had more genes associated with nucleotide metabolism, suggesting a greater need for the gut microbiota to synthesize nucleic acids due to the lack of a sufficient exogenous nitrogen source. While the health consequences to the host are unknown, it is possible that protein consumption leads to alterations in metabolites involved in the inflammatory response, such as uric acid, the precursor of allantoin. These observations warrant future investigation.

## Figures and Tables

**Table 1:** Subject characteristics.

	No PS (No Protein Supplement) (*n* = 17)	PS (Protein Supplement) (*n* = 22)	*p*-Value
Age (years)	33 ± 2 ^1^	32 ± 1	0.84
Weight (lbs)	176 ± 8	173 ± 7	0.79
Height (inches)	67 ± 1	67 ± 1	0.72
BMI (kg/m^2^)	27 ± 1	27 ± 1	0.99
Males	12	14	
Females	5	8	
Total Physical Activity (MET-minutes/week)	7040 ± 1282	12,081 ± 1870	0.03
Total Vigorous Activity (MET-minutes/week)	2535 ± 830	6331 ± 1284	0.02
Upper Body Resistance Exercise Volume (kg/week)	15,743 ± 13,103	31,067 ± 49,323	0.15
Lower Body Resistance Exercise Volume (kg/week)	16,694 ± 19,430	56,464 ± 127,594	0.16
Bristol Scale (arbitrary units)	3.8 ± 0.4	3.3 ± 0.2	0.25
Number of Supplements (count)	0.9 ± 1.5	1.4 ± 2.4	0.45

^1^ Mean ± SEM. MET: Metabolic equivalents.

**Table 2:** Self-reported dietary intake from ASA24.

	No PS (*n* = 17)	PS (*n* = 22)	*p*-Value
Number of Foods	19 ± 2 ^1^	21 ± 2	0.26
Energy (kcal)	2551 ± 429	2452 ± 199	0.84
Protein (g)	117.6 ± 11.8	169.3 ± 17.6	0.02
Protein (g/kg body weight)	1.49 ± 0.14	2.15 ± 0.19	0.009
Calories from protein (%)	20.1 ± 1.5	27.5 ± 1.7	0.003
Carbohydrate (g)	228.3 ± 37.9	239.3 ± 25.9	0.81
Calories from carbohydrates (%)	36.5 ± 2.9	39.3 ± 2.6	0.48
Fiber (g)	18.9 ± 2.1	27.3 ± 3.1	0.03
Total Sugar (g)	93.3 ± 20.7	88.1 ± 13.4	0.84
Kcal from sugar (%)	13.7 ± 1.7	14.1 ± 1.50	0.84
Ratio of protein to carbohydrate (g:g)	0.67 ± 0.11	0.89 ± 0.16	0.26
Fat (g)	109.0 ± 13.7	92.9 ± 9.0	0.33
Total saturated fatty acids (g)	34.1 ± 6.0	29.3 ± 3.2	0.49
Total polyunsaturated fatty acids (g)	24.8 ± 2.7	19.6 ± 2.1	0.14
Total monounsaturated fatty acids (g)	40.6 ± 5.1	35.6 ± 4.2	0.46
Calories from fat (%)	40.8 ± 2.7	33.9 ± 1.9	0.05
Iron (mg)	14.7 ± 1.5	19.5 ± 1.8	0.05
Magnesium (mg)	369 ± 30	533 ± 77	0.03
Potassium (mg)	3064 ± 303	3996 ± 395	0.07
Vitamin C (mg)	120 ± 22	217 ± 49	0.08
Folate, food (mcg)	263 ± 30	375 ± 56	0.09
Intact fruits (whole or cut) of citrus, melons, and berries (cup eq.)	0.065 ± 0.04	0.443 ± 0.15	0.02
Beans and Peas (legumes) computed as protein foods (oz.eq.)	0.132 ± 0.12	0.97 ± 0.4	0.045
Beans and Peas (legumes) computed as vegetables (cup eq.)	0.032 ± 0.03	0.24 ± 0.10	0.045
Healthy Eating Index (HEI)	54.0 ± 13.3	61.8 ± 15.1	0.088
Water (g)	3914 ± 304	4266 ± 441	0.51
Alcohol (g) (14 g = 1 standard drink)	31.4 ± 25.8	1.92 ± 1.1	0.27
Caffeine (mg)	180.3 ± 42.3	156.1 ± 28.5	0.64

^1^ Mean ± SEM.

**Table 3:** Relative abundance of bacterial species that were significantly different between groups and their association with dietary protein and fat.

Bacteria	No PS	PS		Association with Dietary Protein		Association with Dietary Fat	
	Relative Abundance		*p*-Value	Correlation Coefficient	*p*-value	Correlation Coefficient	*p*-Value
p__Actinobacteria							
c__Coriobacteriia	94.9 ± 33.6 ^1^	565.5 ± 158.0	0.008	0.332	0.039	0.513	0.0008
c__Coriobacteriia;o__Coriobacteriales	94.9 ± 33.6	565.5 ± 158.0	0.008	0.332	0.039	0.513	0.0008
c__Coriobacteriia;o__Coriobacteriales;f__Coriobacteriaceae	94.9 ± 33.6	565.5 ± 158.0	0.008	0.332	0.039	0.513	0.0008
c__Coriobacteriia;o__Coriobacteriales;f__Coriobacteriaceae;g__Adlercreutzia	4.3 ± 2.1	54.3 ± 14.1	0.002				
p__Bacteroidetes							
c__Bacteroidia	8923.1 ± 1941.1	16,425.5 ± 1362.1	0.004	0.333	0.038	0.393	0.013
c__Bacteroidia;o__Bacteroidales	8923.1 ± 1941.1	16,425.5 ± 1362.1	0.004	0.333	0.038	0.393	0.013
c__Bacteroidia;o__Bacteroidales;f__Bacteroidaceae;g__Bacteroides;__	181.6 ± 45.4	438.4 ± 81.4	0.0096				
c__Bacteroidia;o__Bacteroidales;f__Rikenellaceae	857.8 ± 269.3	2083.7 ± 265.7	0.003				
c__Bacteroidia;o__Bacteroidales;f__Rikenellaceae;g__	850.2 ± 267.7	2068.4 ± 263.4	0.0026				
p__Firmicutes							
c__Bacilli	30.5 ± 6.8	195.3 ± 42.3	0.0009				
c__Bacilli;o__Lactobacillales	22.6 ± 4.9	136.5 ± 38.1	0.007				
c__Bacilli;o__Turicibacterales	7.1 ± 4.5	58.5 ± 17.6	0.009				
c__Bacilli;o__Turicibacterales;f__Turicibacteraceae	7.1 ± 4.5	58.5 ± 17.6	0.009				
c__Bacilli;o__Turicibacterales;f__Turicibacteraceae;g__Turicibacter	7.1 ± 4.5	58.5 ± 17.6	0.0094				
c__Clostridia	6433.2 ± 1303.2	16,415.6 ± 1526.5	0.00002			0.413	0.009
c__Clostridia;o__Clostridiales	6431.3 ± 1303.0	16,403.7 ± 1522.5	0.00002			0.414	0.009
c__Clostridia;o__Clostridiales;f__	242.7 ± 74.3	739.8 ± 153.0	0.007	0.322	0.046	0.462	0.003
c__Clostridia;o__Clostridiales;f__;g__	242.7 ± 74.3	739.8 ± 153.0	0.0066	0.322	0.046	0.462	0.003
c__Clostridia;o__Clostridiales;f__Clostridiaceae	97.8 ± 23.3	250.4 ± 47.2	0.007				
c__Clostridia;o__Clostridiales;f__Lachnospiraceae	2960.9 ± 639.1	7179.0 ± 686.7	0.0001			0.382	0.016
c__Clostridia;o__Clostridiales;f__Lachnospiraceae;g__	665.6 ± 147.1	1401.9 ± 118.5	0.0004			0.344	0.032
c__Clostridia;o__Clostridiales;f__Lachnospiraceae;g__[Ruminococcus]	190.7 ± 42.5	539.7 ± 80.0	0.0005			0.380	0.017
c__Clostridia;o__Clostridiales;f__Lachnospiraceae;g__[Ruminococcus];s__torques	13.7 ± 6.4	110 ± 32.7	0.008				
c__Clostridia;o__Clostridiales;f__Lachnospiraceae;g__Anaerostipes	17.2 ± 4.9	56.4 ± 12.4	0.0066				
c__Clostridia;o__Clostridiales;f__Lachnospiraceae;g__Coprococcus	371 ± 123	1238.5 ± 191.7	0.0006				
c__Clostridia;o__Clostridiales;f__Ruminococcaceae	2328.5 ± 565.8	6663.8 ± 732.5	0.00004			0.373	0.019
c__Clostridia;o__Clostridiales;f__Ruminococcaceae;__	55.9 ± 10.8	373.2 ± 75.4	0.0004				
c__Clostridia;o__Clostridiales;f__Ruminococcaceae;g__	259.7 ± 74.4	798.3 ± 178.9	0.0097			0.443	0.005
c__Clostridia;o__Clostridiales;f__Ruminococcaceae;g__Faecalibacterium	1372.5 ± 450.4	3488.4 ± 257.8	0.0004				
c__Clostridia;o__Clostridiales;f__Ruminococcaceae;g__Oscillospira	318.6 ± 73.6	822.5 ± 111.8	0.0006			0.445	0.005
c__Clostridia;o__Clostridiales;f__Ruminococcaceae;g__Ruminococcus	314 ± 84	1167.9 ± 275.1	0.0065				
c__Clostridia;o__Clostridiales;f__Veillonellaceae	569.2 ± 122.6	1127.6 ± 147.1	0.00600	0.345	0.031	0.362	0.024
c__Clostridia;o__Clostridiales;f__Veillonellaceae;g__Phascolarctobacterium	171.1 ± 78.3	746.2 ± 151.8	0.0021			0.518	0.0007
c__Clostridia;o__Clostridiales;f__Veillonellaceae;g__Phascolarctobacterium;s__	205.5 ± 79.7	719.7 ± 155.8	0.0062			0.518	0.0007

^1^ Mean ± SEM.

## Data Availability

The data presented in this study are available on request from the corresponding author.
